# Candidate markers for enhanced host response to PRRS have scarce adverse effects on pigs’ growth and production

**DOI:** 10.1186/s40813-024-00379-5

**Published:** 2024-08-21

**Authors:** Houda Laghouaouta, Lorenzo J. Fraile, Joan Estany, Ramona N. Pena

**Affiliations:** Department of Animal Science, University of Lleida-Agrotecnio-CERCA Center, Lleida, Catalonia Spain

**Keywords:** Pigs, Production, PRRS resilience, Selection

## Abstract

**Background:**

Porcine Reproductive and Respiratory Syndrome (PRRS) is one of the most challenging viral diseases that cause substantial economic losses in the pig industry worldwide. The clinical signs of PRRS depend on, among others, the immunomodulatory properties of the PRRS virus strain, farm health status, herd immunity, and host genetics. The high virulence and mutation rate of PRRS virus limit the efficacy of vaccination programs. In recent years, several candidate genetic markers associated with PRRS resilience have been identified, and selective breeding was suggested as an additional approach to control PRRS under field conditions. Even so, it is essential to investigate the effects of these genetic markers on pigs’ productivity. Our study aimed to assess the association between seven previously reported candidate genetic markers for host response to PRRS (rs80800372 in *GBP1*, rs340943904 in *GBP5*, rs322187731 in *GBP6*, rs1107556229 in *CD163*, rs338508371 in *SGK1*, rs80928141 in *TAP1*, and a 275-bp insertion in the promoter of *MX1*) and production traits in pigs under non-challenging conditions.

**Results:**

About 600 high-health Duroc pigs were genotyped for the selected genetic markers and their effects on production traits (live body weight, carcass weight, backfat thickness, intramuscular fat content and composition) were assessed using a linear model. The genetic markers GBP5_rs340943904, GBP6_rs322187731, CD163_rs1107556229, and the 275-bp insertion at the promoter of *MX1* showed no relevant associations with growth and carcass traits at slaughter. Regarding GBP1_rs80800372 (WUR1000125), the favourable G allele for PRRS resilience displayed significant additive effects on backfat thickness (+ 1.18 ± 0.42 mm; *p* = 0.005) and lean content (-1.72 ± 0.56%; *p ≤* 0.01) at slaughter. In addition, the genetic markers SGK1_rs338508371 and TAP1_rs8092814 were associated with the palmitoleic content in *gluteus medius*, without affecting the total of the monounsaturated fatty acids.

**Conclusions:**

Our results indicate that genetic markers for PRRS resilience have no relevant effects on growth and carcass traits in pigs reared under non-challenging conditions, except for GBP1_rs80800372 where the favourable allele for PRRS response has a negative impact on lean content. Therefore, since the effects of GBP1_rs80800372 were attributed to the causal variant GBP5_rs340943904, it seems beneficial to select pigs for the genetic marker at *GBP5* instead of *GBP1*. Overall, pigs might be selected for enhanced PRRS resilience without compromising their overall productivity.

**Supplementary Information:**

The online version contains supplementary material available at 10.1186/s40813-024-00379-5.

## Background

In the context of animal production, resilience can be defined as the ability of an animal to withstand and recover from various stressors or challenges without experiencing significant negative impacts on health or productivity. These stressors can include a wide range of factors, such as changes in environmental conditions, exposure to infectious diseases, nutritional deficiencies, or other management or husbandry practices. Resilience can be affected by a variety of factors, including genetics. In relation to pig production, resilience to infections, and particularly to the porcine reproductive and respiratory syndrome (PRRS), has been investigated in depth [1]. Given the difficulty in triggering robust and effective protection through vaccines, selection for animals with a better tolerance to the infection has been proposed as a complementary strategy [2]. Indeed, the genetic component of response to PRRS virus infections has been shown in many studies [3], involving structural [4–6] and functional [7–9] genomic approaches. Despite all the efforts, so far, only a small number of DNA markers have been proposed to enhance resilience to PRRS virus infections, and an even smaller number have been validated in different populations.

One of the best-studied markers for response to PRRS virus infections is the WUR10000125 (rs80800372) marker, located in pig chromosome (SSC) 4, in the 3’UTR of the guanylate binding protein (GBP) 1 gene (*GBP1*). This marker shows an additive effect over viremia levels and weight gain during outbreaks in nursery and growing pigs and has been validated in different populations [1, 10]. In contrast, the effect that this marker might have on the reproductive outcome of sows is less consistent, both in positive [11] and negative [5, 12] scenarios. However, the causal effect of this SSC4 region is believed to be in the rs340943904 marker in *GBP5*, situated about 136 kb upstream from the WUR10000125 marker [13]. Given their proximity, these two markers exhibit a partial, but not complete, linkage disequilibrium, estimated in the range of r^2^ = 0.7-1.0 in many commercial lines [13–15] and as low as r^2^ = 0.21 in Korean native pigs [16] or 0.13 in Italian breeds (Cinta Senese) [17]. The GBP cluster at SSC4 also includes the *GBP6* gene [15]. Mutations in this gene have been linked to changes in blood cell counts [18], with putative implications in immune response to pathogens. Other DNA markers have been successfully tested for clinical and immune response outcomes following PRRS outbreaks but have rarely been validated in more than one population. These include a 275-bp insertion in the *MX1* promoter [19] and single nucleotide polymorphisms (SNPs) variants in the *CD163* (rs1107556229), *SGK1* (rs338508371), and *TAP1* (rs80928141), which were associated with reproductive outcomes in PRRS-infected sows [11, 20].

Once piece of information that strikes from the previous reports is that the frequency of the favourable allele is much lower in commercial [14] and Asian [16] populations than in some European [17, 21] local breeds. One might argue that selection for higher productivity might have worked against the presence of these variants. That would imply that there might be a negative impact of the resilience variants on productivity traits, at least in high-health nucleus farms. Few studies so far have questioned the effect of these variants on productivity. As an example, the favourable G allele (under PRRS challenge) of WUR10000125 did not show consistent detrimental effects on economic traits under non-challenging conditions [22], but a negative effect for average daily gain was described in non-challenged Duroc pigs [23]. Therefore, the aim of the present study is to further investigate on the possible relationship between seven PRRS-resilience candidate markers and production traits under non-challenging PRRS conditions.

## Methods

### Animals and phenotypes

About 600 commercial Duroc pigs from a high-health status farm were used in this study. Pigs were reared in seven fattening batches under the same conditions with *ad libitum* access to commercial diets from about 75 days of age to slaughter (210 days, SD 8). No major diseases were reported during the fattening period (PRRS-free). At 120 (SD 7), 180 (SD 5), and 207 (SD 8) days of age, pigs were weighed and their backfat and loin thickness were ultrasonically measured at 5 cm off the midline at the position of the last rib (Piglog 105; Frontmatec). At slaughter (210 days, SD 8), carcass was weighed and carcass backfat and loin thickness at 6 cm off the midline between the third and fourth last ribs were estimated by an ultrasound automatic scanner (AutoFOM, Frontmatec Group, Denmark). In addition, the intramuscular fat (IMF) content and fatty acid composition of the *gluteus medius* muscle were quantified by gas chromatography as described in [24]. The fatty acids profile was expressed as the percentage of each fatty acid relative to the total fatty acid content. Then, the proportions of saturated (SFA = C14:0 + C16:0 + C18:0 + C20:0), monounsaturated (MUFA = C16:1n-7 + C18:1n-7 + C18:1n-9 + C20:1n-9), and polyunsaturated (PUFA = C18:2n-6 + C18:3n-3 + C20:2n-6 + C20:4n-6) fatty acids were calculated.

### DNA isolation and genotyping of genetic markers for the host response to PRRS

Seven previously reported genetic markers for the host response to PRRS were analysed in this study (Table 1). Genomic DNA was isolated using standard phenol/chloroform protocols [25]. Then, all pigs were genotyped for the selected genetic markers (Table S1). The genetic marker WUR1000125 (rs80800372) at *GBP1* was genotyped by real-time PCR and allelic discrimination with primers and conditions as described by [26]. Briefly, PCR was performed in a QuantStudio 3 thermocycler (Applied Biosystems, Thermo Scientific) in a final volume of 5 µl containing 1× TaqMan Universal PCR Master Mix, 1× primer and probe set, and about 10 ng of the genomic DNA. PCR started with an initial fluorescence detection at 60 ºC for 1 min, denaturation at 95 ºC for 10 min, 40 cycles of 95 ºC for 10 s and 60 ºC for 1 min, followed by a final fluorescence detection at 60 ºC for 1 min. Genetic markers at *GBP5* (rs340943904), *GBP6* (rs322187731), *CD163* (rs1107556229), *SGK1* (rs338508371), and *TAP1* (rs80928141) were genotyped using real-time PCR and high-resolution melt protocols. PCR was performed in a QuantStudio 3 thermocycler (Applied Biosystems, Thermo Scientific) in a final volume of 5 µl containing 1× MeltDoctor HRM Master Mix (ThermoScientific), about 10 ng of the genomic DNA and 0.4 µM of each primer. PCR started with a denaturation at 95 ºC for 10 min, 40 cycles of 95 °C for 15 s and 60 °C for 30 s, followed by a high-resolution melting curve starting with denaturation at 95 °C for 15 s, annealing at 60 °C for 1 min, and a slow ramp at 0.015 °C/s to 95 °C. The resulting melting curves were compared, and genotypes were assigned using the high-resolution melt software v3.1 (Applied Biosystems, Thermo Scientific). The 275-bp insertion within the promoter of *MX1* was genotyped by end-point PCR and agarose gel electrophoresis. PCR was performed in a final volume of 15 µl containing 1× buffer, 2 mM of MgCl_2_, 0.16 mM of dNTPs, 0.32 µM of each primer, 0.75 U of Taq polymerase (Bioline), and 30 ng of genomic DNA. All primers and probes are provided in Table S1. Additionally, all pigs were genotyped for two markers associated with lipid content and composition in this breed, located at the leptin receptor (*LEPR*; rs709596309) and the stearoyl-CoA desaturase (*SCD*; rs80912566) genes as described in [27] and [28], respectively.

**Table 1:** Selected genetic markers for the porcine reproductive and respiratory syndrome

Gene	Marker	Position^1^	Polymorphism	Favourable allele^2^	Reference
*GBP1*	rs80800372	4:127441677	A > G	G	[4, 23]
*GBP5*	rs340943904	4:127301202	G > T	T	[11, 13]
*GBP6*	rs322187731	4:127190259	A > G	G	[18]
*CD163*	rs1107556229	5:63325006	G > A	G	[11, 34]
*SGK1*	rs338508371	1:29753070	C > A	C	[20]
*TAP1*	rs80928141	7:25068055	C > T	T	[20]
*MX1*	-547ins + 275	13:204845962	Indel + 275 bp	Insertion	[11, 35]

^1^Position in the pig genome assembly *Sscrofa 11.1*

^2^Favourable allele for the response to the porcine reproductive and respiratory syndrome

### Statistical analyses

The number of the analysed individuals varied among analyses due to missing genotypes or phenotypes, ranging from 559 to 618. The effects of each candidate genetic marker on production traits (live body weight, backfat and loin thickness at 120, 180 and 207 days, as well as carcass weight, backfat and loin thickness, lean content, and IMF content and composition at slaughter) were estimated separately using single-marker analyses. Each model included the fattening batch (7 levels) and the genotype for *LEPR* (3 levels), *SCD* (3 levels), and a candidate marker for PRRS (3 levels), with the age at measurement as a covariate. Since the genetic markers at *SCD* and *LEPR* were previously associated with lipid content and composition, they were added to the model to adjust the analysed phenotype for their effects. In addition, the IMF content was added as a covariate for fatty acid composition traits. Multiple pairwise comparisons across genotypes for PRRS markers were performed using the Tukey HSD test. Additive and dominant effects were tested by replacing the genotype with two covariates coded as (-1, 0, 1) and (0, 1, 0), respectively. All statistical analyses were performed using JMP Pro v16 (SAS Institute Inc, Cary, NC, USA). For each test, *p*-values were adjusted using Bonferroni correction. Since for each trait seven genetic markers were tested, the significance was set at 0.007.

### Linkage disequilibrium

The linkage disequilibrium (LD) between GBP1_rs80800372, GBP5_rs340943904, and GBP6_rs322187731 at SSC4 was measured as the square of the correlation coefficient (r^2^) between the alleles at two of these markers using the PLINK v1.9 software [29].

## Results

### Allelic and genotypic frequencies of the candidate markers for the host response to PRRS

The allelic and genotypic frequencies of the analysed genetic markers for the host response to PRRS are given in Table 2. The minor allele frequencies ranged from 0.15 for SGK1_rs338508371 to 0.35 for CD163_rs1107556229. Except for SGK1_rs338508371 and CD163_rs1107556229, the reported favourable alleles for PRRS resilience had the lowest allelic frequencies (Tables 1 and 2).

**Table 2:** Allelic and genotypic frequencies of the studied genetic markers for porcine reproductive and respiratory syndrome

Gene	Marker	Position	Minor allele	MAF	*N*	11	12	22
*GBP1*	rs80800372	4:127441677	G	0.18	618	0.67	0.30	0.03
*GBP5*	rs340943904	4:127301202	T	0.26	578	0.54	0.41	0.05
*GBP6*	rs322187731	4:127190259	G	0.23	559	0.59	0.37	0.04
*CD163*	rs1107556229	5:63325006	A	0.35	579	0.43	0.44	0.13
*SGK1*	rs338508371	1:29753070	A	0.15	590	0.77	0.16	0.07
*TAP1*	rs80928141	7:25068055	T	0.26	578	0.55	0.39	0.07
*MX1*	-547ins + 275	13:204845962	Insertion	0.20	563	0.63	0.33	0.04

MAF: minor allele frequency; N: Number of analysed animals for each genetic marker; 1 and 2 refer to the major and minor alleles, respectively

### Association between candidate markers for the host response to PRRS and production

A summary of the production traits that came out associated with the candidate markers for the host response to PRRS is given in Table 3. Within the GBP gene family, no significant differences were found between the GBP5_rs340943904 and GBP6_rs322187731 genotypes for the analysed production traits (Table 3, Table S2, and Table S3). In contrast, significant differences were detected between the GBP1_rs80800372 genotypes in backfat thickness at slaughter (*p =* 0.004) and lean content (*p* = 0.002) (Table 4). The beneficial G allele for PRRS resilience had a significant additive effect on backfat thickness at slaughter (+ 1.18 ± 0.42 mm; *p* ≤ 0.01), intramuscular fat content (+ 1.53 ± 0.53%; *p* ≤ 0.01), and lean content (-1.72 ± 0.56%; *p* = 0.005) (Table 4). No association was found between the GBP1_rs80800372 genotypes and the intramuscular fatty acid composition (Table S4). The linkage disequilibrium (r^2^) between the genetic markers at *GBP1*-*GBP5*, *GBP1*-*GBP6*, and *GBP5*-*GBP6* were 0.56, 0.60, and 0.81, respectively.

**Table 3:** Associated production traits with the candidate markers for the host response to the porcine reproductive and respiratory syndrome

Gene	Marker	Associated production traits
*GBP1*	rs80800372	Backfat thickness at slaughter and lean content
*GBP5*	rs340943904	-
*GBP6*	rs322187731	-
*CD163*	rs1107556229	-
*SGK1*	rs338508371	Palmitoleic content
*TAP1*	rs80928141	Palmitoleic content
*MX1*	-547ins + 275	Body weight and backfat thickness at 120 days

**Table 4:** Least square means for the analysed growth and carcass traits by the GBP1_rs80800372 (A > G) genotype

Trait	AA	AG	GG	*p*	Additive effect^1^			Dominant effect	
	(*N* = 414)	(*N* = 185)	(*N* = 19)		a	*p*		d	*p*
**Live measurements at 120 days**									
Body weight (Kg)	61.9 ± 0.40	61.9 ± 0.57	61.6 ± 1.68	ns	**-**	**-**		**-**	**-**
Backfat thickness (mm)	11.1 ± 0.12	11.3 ± 0.17	11.8 ± 0.50	ns	**-**	**-**		**-**	**-**
Loin thickness (mm)	40.4 ± 0.20	40.2 ± 0.28	41.7 ± 0.93	ns	**-**	**-**		**-**	**-**
**Live measurements at 180 days**					**-**	**-**		**-**	**-**
Body weight (Kg)	107.5 ± 0.54	108.2 ± 0.79	109.8 ± 2.31	ns	**-**	**-**		**-**	**-**
Backfat thickness (mm)	17.9 ± 0.19	17.9 ± 0.27	19.9 ± 0.80	**ns**	-	**-**		-	**-**
Loin thickness (mm)	45.1 ± 0.21	44.7 ± 0.31	44.5 ± 0.91	ns	**-**	**-**		**-**	**-**
**Live measurements at 207 days**									
Body weight (Kg)	122.3 ± 0.60	123.1 ± 0.86	124.7 ± 2.53	ns	**-**	**-**		**-**	**-**
Backfat thickness (mm)	20.6 ± 0.21	20.8 ± 0.31	22.3 ± 0.90	ns	**-**	**-**		**-**	**-**
Loin thickness (mm)	48.6 ± 0.24	48.4 ± 0.35	49.3 ± 1.02	ns	**-**	**-**		**-**	**-**
**Carcass measurements**									
Carcass weight (Kg)	93.6 ± 0.50	94.3 ± 0.71	96.4 ± 2.09	ns	**-**	**-**		**-**	**-**
Backfat thickness (mm)	22.3 ± 0.20^b^	22.9 ± 0.28^ab^	24.6 ± 0.83^a^	**0.004**	+ 1.18 ± 0.42	**0.005**		-	-
Loin thickness (mm)	45.5 ± 0.39	44.8 ± 0.55	42.9 ± 1.62	ns	**-**	**-**		**-**	**-**
IMF (% dry matter)	15.2 ± 0.25	15.4 ± 0.36	18.2 ± 1.04	**ns**	+ 1.53 ± 0.53	**0.004**		−1.25 ± 0.62	ns
Lean content (%)	44.2 ± 0.26^a^	43.3 ± 0.37^ab^	40.8 ± 1.10^b^	**0.002**	−1.72 ± 0.56	**0.002**		0.77 ± 0.65	ns

^1^the substitution effect of A for G

Bold indicates statistical significance (*p*-value lower than the Bonferroni threshold 0.007)

Within each trait, means with different superscripts (^a^,^b^) indicate significant differences (*p*-value lower than the Bonferroni threshold 0.007) between the genotypes

IMF: intramuscular fat, ns: not significant

The genetic marker CD163_rs1107556229 was associated with any of the studied production traits (Table S5). The remaining genetic markers, SGK1_rs338508371, TAP1_rs80928141, and the 275-bp insertion at the promoter of *MX1* had minor effects over production traits (Table 3). For SGK1_rs338508371, no significant associations were detected with growth and carcass traits (Table S6). This genetic marker was only associated with the palmitoleic (C16:1n-7) content in *gluteus medius* (*p* = 0.0003). AA pigs showed a higher C16:1n-7 content (4.05 ± 0.10%) compared to AC (3.69 ± 0.07%) and CC (3.62 ± 0.03%) pigs. The A allele had an additive effect of + 0.21 ± 0.05% (*p* = 0.00006) on the content of this fatty acid (Table S6), yet no significant differences were detected for the total monounsaturated fatty acids. Similarly to SGK1_rs338508371, TAP1_rs8092814 was associated with C16:1n-7 content in *gluteus medius* (*p* = 0.006), with an additive effect of the T allele of 0.17 ± 0.05% (*p* = 0.001) but without affecting the total of the monounsaturated fatty acids (Table S7). Finally, the 275-bp insertion at the promoter of *MX1* was associated with live body weight (*p* = 0.0006) and backfat thickness (*p* = 0.005) at 120 days of age. However, thereafter, and up to the end of the fattening period, no differences were found across the *MX1* genotypes in the studied production traits (Table S8).

## Discussion

To date, attempts to control PRRS have not been completely successful. The success of vaccination programs has been limited by the PRRS virus’s high virulence and mutation rate [30], while eradication measures such as whole herd depopulation/repopulation, test and removal, and herd closure are economically unfeasible. Hence, complementary strategies to control PRRS are needed to reduce its economic losses, improve pigs’ health and welfare, and therefore, set up a more sustainable and efficient pig farming. The genetic component of the host response to PRRS virus infection has been well-evidenced, indicating variation in host immune responses and disease outcomes. Over time, several candidate genetic markers associated with the host response to PRRS have been identified [1, 31]. Therefore, selective breeding has been suggested as a complementary effort to control PRRS. However, owing to the well-known antagonism between resilience and production [32], selection for enhanced PRRS resilience may adversely affect production traits and the overall profitability of the swine industry. Thus, it is essential to evaluate the effects of the genetic markers for the host response to PRRS on production traits under non-challenging conditions prior to their implementation in pig breeding programs. To do so, this work assessed the effects of a panel of seven previously reported candidate genetic markers for the host response to PRRS on 24 production traits in pigs reared in a high-health situation (PRRS-free) under no major challenging conditions.

As expected, most of the favourable alleles for the host response to PRRS had the lowest allelic frequencies. One possible explanation is that these favourable alleles for PRRS resilience are negatively associated with economically important traits such as production and, consequently, have been unintentionally selected against. Another possible explanation may be that, apart from their importance for PRRS resilience, they exhibit no relevant positive effects on traits under selection and their frequency has not been under any selection pressure. The low frequency of the favourable alleles limited the number of homozygous pigs in our study (Table 4 and Supplementary Tables S2 to S8), which might constrain power in the statistical analyses.

As mentioned previously, a major QTL at SSC4 was associated with the host response to PRRS, explaining about 15% of the genetic variance of viremia levels during PRRS outbreak. This QTL harbours five members from the GPB family (*GBP1*,* GBP2*,* GBP4*,* GBP5*, and *GBP6*). Initially, the genetic marker rs80800372 at the 3´ UTR region of the *GBP1* gene was used as a tag variant to capture the effects of this QTL [4, 10]. Effects of this marker on the host response to PRRS have been validated across several genetic lines challenged with American and European PRRS virus strains, all indicating that the G allele is favourable for PRRS resilience [4, 10, 23]. Yet, in a recent allele-specific expression analysis, the causality of this QTL was attributed to the genetic marker GBP5_rs340943904 [13]. Another candidate gene for the host response to PRRS within the GBP gene family is *GBP6* as its expression is up-regulated in pulmonary alveolar macrophages and correlated with viremia levels after PRRS virus infection [8]. The genetic marker GBP6_rs322187731 was associated with immune-related traits, suggesting its possible implication in regulating the host response to infection [18]. In contrast with the numerous studies on the host response to PRRS, there is scarce and controversial information about the effects of the markers within the GBP cluster on production traits. For instance, effects of GBP1_rs80800372 on growth rate are not consistent across studies. AG pigs showed higher growth rates than AA pigs during PRRS virus infection in several studies [4, 10]. In contrast, the opposite effect was observed in PRRS virus-free environments [23], which was not confirmed in other studies [22]. In the current work, no significant associations were detected between GBP1_rs80800372 and growth traits (live body and carcass weights) nor with the fatty acid composition of *gluteus medius*. Yet, this genotype was associated with carcass fat content. The beneficial G allele for PRRS resilience was related to greater backfat thickness, greater intramuscular fat content, and lower lean content. Effects of GBP1_rs80800372 on fat content may be beneficial for some production systems, for instance the production of pork for dry-curing or other artisanal processing products. Conversely, its effect on lean content might have unwanted consequences on carcass price, at least in Europe. These results contrast with the lack of negative associations between GBP1_rs80800372 and productivity under non-challenging conditions in Italian pigs [17] and other commercial lines [22].

The moderate LD between GBP1_rs80800372 and the other two markers on the GBP cluster (0.56–0.60) might explain the total lack of association of the latter with the traits on study. Overall, these results are in agreement with those reported in Italian pigs for GBP5_rs340943904, while to the best of our knowledge no data is available regarding the relationship between GBP6_rs322187731 and productivity. Taken together the results from the GBP cluster, the two variants GBP5_rs340943904 and GBP6_rs322187731 seem to have no relevant effects on the overall production in pigs reared under non-challenging conditions, whereas GBP1_rs80800372 seems to adversely affect the lean content. Consequently, and given that the causality of PRRS resilience was attributed to GBP5_rs340943904 rather than GBP1_rs80800372 [13], selective breeding for the favourable T allele at the *GBP5* marker could improve the host resilience to PRRS without compromising production. This strategy comes with a caveat: that it is conditional to the LD between the *GBP1* and *GBP5*, which, as stated above, varies across genetic lines.

The other studied candidate markers for the host response to PRRS had no relevant adverse effects on the production traits. The genetic marker rs1107556229 is a splice region variant at the *CD163* gene. This gene encodes a cysteine-rich scavenger receptor for PRRS virus, which is necessary and sufficient for macrophage infection by the virus [33]. Knockout of *CD163* resulted in resistance against PRRS virus, but deleting *CD163* may impair essential physiological functions [33]. Naturally occurring mutations within *CD163* were further associated with the host response to PRRS virus infection such as viremia and antibody levels [7, 34, 35] and abortion rates [11]. In our study, CD163_rs1107556229 had no significant effects on the studied growth and carcass traits, indicating that its inclusion in breeding programs may be possible without adversely affecting the overall productivity.

The genetic markers SGK1_rs338508371 and TAP1_rs80928141 were previously associated with reproductive traits after PRRS infection in pigs [20]. The C allele for SGK1_rs338508371 and the T allele for TAP1_rs80928141 were associated with better reproductive performance during a PRRS outbreak. Therefore, selective breeding for these alleles was suggested to maintain stable reproductive performance despite PRRS virus infection. In our population, neither marker had relevant effects on growth and carcass traits, despite their association with the palmitoleic (C16:1n-7) content in *gluteus medius*. The favourable allele for SGK1_rs338508371 was associated with lower palmitoleic content, whereas the favourable T allele for *TAP1* was associated with higher palmitoleic content. The magnitude of change represents a variation of about 5% of the total palmitoleic content, with no consequence in the total monounsaturated fatty acids content. Therefore, no impact on pork quality is expected as consequence of these two markers. The last studied genetic marker was a 275-bp insertion at the promoter of the *MX1* gene. *MX1* encodes a guanosine triphosphate-metabolizing protein long known for its antiviral activity in several species [36]. In pigs, *MX1* expression was involved in the innate host response against PRRS virus [37]. The insertion of a 275-bp fragment of a SINE element at the promoter of *MX1* was associated with better resistance to PRRS virus in vitro [11, 19] and in vivo [11]. In our hands, this polymorphism showed no relevant effects on the production traits of pigs at slaughter that would guard against its use on selective breeding.

## Conclusions

Taken together, effects of three candidate genetic markers within the major QTL at SSC4 for the host PRRS response were assessed on production traits. Although, the genetic marker GBP1_rs80800372 seems to adversely affect the lean content, the causal variant GBP5_rs340943904 had no negative effects on any of the studied production traits. Therefore, selective breeding for the favourable allele for PRRS resilience at GBP5_rs340943904 may be possible without compromising the overall production under non-challenging conditions. Moreover, other candidate genetic markers for the host response to PRRS such as CD163_rs1107556229, SGK1_rs338508371, TAP1_rs80928141, and a 275-bp insertion at *MX1* had no relevant drawbacks on pigs’ production under non-challenging conditions. Some significant associations were found between the selected genetic markers and the production traits at the beginning of the fattening period, yet they were not consistent at later times or slaughter. Therefore, selective breeding for the favourable alleles for PRRS resilience at these variants may be applied as an additional effort to control PRRS, along with vaccination and adequate biosecurity measures. However, further validations with larger datasets and different genetic lines are needed to corroborate our results. Besides, it would be important to evaluate the effects of genetic markers for the host response to PRRS on other important traits, such as reproductive performance.

### Electronic supplementary material

Below is the link to the electronic supplementary material.

**Supplementary Material 1**: **Table S1**.Description of primers and probes used for genotyping the studied genetic markers. ^1^Position in the pig genome assembly *Sscrofa 11.1*. **Table S2** Least square means for the analysed production traits by the GBP5_rs340943904 (G > T) genotype. SFA: saturated fatty acids, C16:0: palmitic acid, C18:0: stearic acid, MUFA: monounsaturated fatty acids, C16:1n-7: palmitoleic acid, C18:1 refers to the sum of the vaccenic (C18:1n-7) and the oleic (C18:1n-9) acids, PUFA: polyunsaturated fatty acids, C18:2n-6: linoleic acid, C18:3n-3: linolenic acid, C20:4n-6: arachidonic acid, ns: not significant. **Table S3**, Least square means for the analysed production traits by the GBP6_rs322187731 (A > G) genotype. SFA: saturated fatty acids, C16:0: palmitic acid, C18:0: stearic acid, MUFA: monounsaturated fatty acids, C16:1n-7: palmitoleic acid, C18:1 refers to the sum of the vaccenic (C18:1n-7) and the oleic (C18:1n-9) acids, PUFA: polyunsaturated fatty acids, C18:2n-6: linoleic acid, C18:3n-3: linolenic acid, C20:4n-6: arachidonic acid, ns: not significant. **Table S4** Least square means for the fatty acid composition (%) of *gluteus medius* by the GBP1_rs80800372 (A > G) genotype. SFA: saturated fatty acids, C16:0: palmitic acid, C18:0: stearic acid, MUFA: monounsaturated fatty acids, C16:1n-7: palmitoleic acid, C18:1 refers to the sum of the vaccenic (C18:1n-7) and the oleic (C18:1n-9) acids, PUFA: polyunsaturated fatty acids, C18:2n-6: linoleic acid, C18:3n-3: linolenic acid, C20:4n-6: arachidonic acid, ns: not significant. **Table S5** Least square means for the analysed production traits by the CD163_rs1107556229 (G > A) genotype. SFA: saturated fatty acids, C16:0: palmitic acid, C18:0: stearic acid, MUFA: monounsaturated fatty acids, C16:1n-7: palmitoleic acid, C18:1 refers to the sum of the vaccenic (C18:1n-7) and the oleic (C18:1n-9) acids, PUFA: polyunsaturated fatty acids, C18:2n-6: linoleic acid, C18:3n-3: linolenic acid, C20:4n-6: arachidonic acid, ns: not significant. **Table S6**^1^ the substitution effect of C for A. SFA: saturated fatty acids, C16:0: palmitic acid, C18:0: stearic acid, MUFA: monounsaturated fatty acids, C16:1n-7: palmitoleic acid, C18:1 refers to the sum of the vaccenic (C18:1n-7) and the oleic (C18:1n-9) acids, PUFA: polyunsaturated fatty acids, C18:2n-6: linoleic acid, C18:3n-3: linolenic acid, C20:4n-6: arachidonic acid, ns: not significant. Bold indicates statistical significance. Within each trait, means with different superscripts (^a^,^b^) indicate significant differences (*p*-value lower than the Bonferroni threshold 0.007) between the genotypes. **Table S7** Least square means for the analysed production traits by the TAP1_rs80928141 (C > T) genotype. ^1^ the substitution effect of C for T. SFA: saturated fatty acids, C16:0: palmitic acid, C18:0: stearic acid, MUFA: monounsaturated fatty acids, C16:1n-7: palmitoleic acid, C18:1 refers to the sum of the vaccenic (C18:1n-7) and the oleic (C18:1n-9) acids, PUFA: polyunsaturated fatty acids, C18:2n-6: linoleic acid, C18:3n-3: linolenic acid, C20:4n-6: arachidonic acid, ns: not significant. Bold indicates statistical significance. Within each trait, means with different superscripts (^a^,^b^) indicate significant differences (*p*-value lower than the Bonferroni threshold 0.007) between the genotypes. **Table S8** Least square means for the analysed production traits by the genotypes of the 275-bp insertion at *MX1*. ^1^The additive effect of the 275-bp insertion. SFA: saturated fatty acids, C16:0: palmitic acid, C18:0: stearic acid, MUFA: monounsaturated fatty acids, C16:1n-7: palmitoleic acid, C18:1 refers to the sum of the vaccenic (C18:1n-7) and the oleic (C18:1n-9) acids, PUFA: polyunsaturated fatty acids, C18:2n-6: linoleic acid, C18:3n-3: linolenic acid, C20:4n-6: arachidonic acid, ns: not significant. Bold indicates statistical significance. Within each trait, means with different superscripts (^a^,^b^) indicate significant differences (*p*-value lower than the Bonferroni threshold 0.007) between the genotypes.

## Data Availability

Genotypes for the seven candidate genetic markers for the host response to PRRS and the associated phenotypes are available from the CORA Research Data Repository (Provisional link) https://dataverse.csuc.cat/privateurl.xhtml? token=cd35730a-0bbc-4ac0-a307-f0964c38ff29.
